# The first complete mitochondrial genome of *Dermestes dimidiatus* ab. *rosea* Kusnezova and its phylogenetic implications for the superfamily Bostrichoidea

**DOI:** 10.1080/23802359.2020.1840931

**Published:** 2020-12-24

**Authors:** Yuning Wang, Yue Zhang, Shuhua Wei, Farman Ullah, Youting Pang, Chengcai Ma, Zhihong Li

**Affiliations:** a Department of Entomology, College of Plant Protection, China Agricultural University, Beijing, China; b Ningxia Academy of Agriculture and Forestry Sciences, Plant Protection Institute, Yinchuan, Ningxia, China; c College of Life Sciences, North Minzu University, Yinchuan, Ningxia, China

**Keywords:** *Dermestes dimidiatus* ab. rosea Kusnezova, mitochondrial genome, phylogenetic analysis, Bostrichoidea

## Abstract

In this study, the complete mitochondrial genome of *Dermestes dimidiatus* ab. *rosea* was characterized using next-generation sequencing, and the phylogenetic relationships of superfamily Bostrichoidea were established. The results showed that the mitochondrial genome of *D. dimidiatus* ab. *rosea* was 16,073 bp in size, and it contained thirteen protein-coding genes (PCGs), twenty-two transfer RNA genes (tRNAs), two ribosomal RNA genes (rRNAs), and a control region. The composition of the whole mitochondrial genome of this species was 41.3% A, 13.5% C, 9.3% G, and 35.9% T, which had high A + T content (77.2%). Phylogenetic relationships of the superfamily Bostrichoidea showed that *D. dimidiatus* ab. *rosea* and *Dermestes tessellatocollis* formed in a clade that was a sister group to (*Dermestes maculatus* + *Dermestes frischii*), indicating that Dermestidae was a monophyletic group. This is the first report of a complete mitochondrial genome of *D. dimidiatus* ab. *rosea* and preliminary study of Bostrichoidea mitochondrial genome, which is of great significance for the molecular identification of this species and the enrichment of mitochondrial genome database.

*Dermestes dimidiatus* ab. *rosea* Kusnezova is a black and rose colored hairy beetle which belongs to superfamily Bostrichoidea. It distributes in former Soviet Union and Mongolia, as well as China including four provinces (Heilongjiang, Inner Mongolia, Qinghai, and Gansu) and three Autonomous Regions (Ningxia, Xinjiang, and Tibet) (Zhang et al. 2016). *D. dimidiatus* ab. *rosea* can damage the stored raw skin of animal. Furthermore, it is a kind of saprophagous insect that is important to maintain the prairiebalance and stability of the ecosystem by eating animal carcasses, degrading feces, and cadavers. But up to now, we found minimal molecular data in GenBank for superfamily Bostrichoidea, even some of the mitochondrial gene sequences were not complete. In this study, complete mitochondrial genome data of *D. dimidiatus* was sequenced, which could be useful in further studies for infestation diagnosis, evolution, and phylogeny research within superfamily Bostrichoidea.

Adults *D. dimidiatus* ab. *rosea* were collected from Gaoshawo (37°99′N, 106°21′E) in Ningxia in June 2018. These samples were identified as *D. dimidiatus* ab. *rosea,* according to Zhang’s monographs (2016). Adults specimen of *D. dimidiatus* ab. *rosea* were preserved at −4 °C in 100% ethyl alcohol at Plant Quarantine and Invasion Biology Lab, China Agricultural University, Beijing. We used an adult beetle with the elytra removed and finished the DNA extraction using the DNeasy DNA Extraction kit (QIAGEN).The Berry Genomics sequencing company (Beijing, China) finished the genomic DNA library preparation and sequencing. Genomic DNA was fragmented with Bioruptor to an average insert size of 450 bp and sequenced on an Illumina Hiseq 2500 (Yang et al. 2018). By using the Geneious R10.0, we picked the complete mitochondrial genome sequence of *D. dimidiatus* ab. *rosea*. Using MITOS (Bernt et al. 2013) and tRNA-scan (Chan and Lowe 2019), we predicted the position and direction of thirteen PCGs, two rRNA genes, and twenty-two tRNA genes with the following parameters: Reference=“RefSeq 63 Metazoa” and Genetic Code=“5 Invertebrate”. To explore the phylogenetic relationship of the superfamily Bostrichoidea, we chose fourteen species belonging to Bostrichoidea. The whole mitochondrial genome sequence of *Cryptolestes ferrugineus*, which belongs to Cleroidea, was used as the outgroup. For each gene from fifteen species, sequences were aligned using ClustalW with the default parameters implemented in MEGA5.0 (Tamura et al. 2011). Aligned sequences for each gene were concatenated using SequenceMatrix v1.7 (Vaidya et al. 2011). After aligning, cutting, and assembling sequences, two datasets were used in subsequent phylogenetic analyses: (1) PCG123: all three codon positions of the PCGs (total 10,662 nucleotides); (2) PCG123 and 2 rRNAs: all three codon positions of PCGs and rRNA (total 12,177 nucleotides). Bayesian analysis was applied using the MrBayes on XSEDE v3.2.6v (Ronquist and Huelsenbeck 2003). Twoindependent runs with four simultaneous chains were run for 10,000,000 generations. Samples were drawn every 1,000 steps, with the first 25% discarded as burn-in, the maximum likelihood analysis was run using the IQ TREE on XSEDE v1.6.10 (Nguyen et al. 2015). PartitionFinder, IQ Tree, and MrBayes were run through the CIPRES Science Gateway V3.3 (Miller et al. 2016). The bootstrap supports ≥75% for ML analysis, and posterior probabilities ≥0.90 for Bayesian analysis of each node were considered as reliable support values.The complete mitochondrial genome of *D.dimidiatus ab. rosea* (Genbank:MN245298) was 16,073 bp in length, containing thirteen PCGs, twenty-two tRNA genes, two rRNA genes and a large non-coding region (Wolstenholme, 1992; Boore et al., 1998), and there was an asymmetric nucleotide composition (41.3% A, 13.5% C, 9.3% G, and 35.9% T). There were three types of start codons (ATT, ATA, and ATG) in the mitochondrial genome of *D. dimidiatus ab. rosea* and three types of stop codons (TAA, TAG, and T**) for translation termination. Except for trnS1, all tRNA sequences could fold into the typical cloverleaf secondary structure. Particularly, the length of *trnS1*’s DHU arm was 0 bp, which was supported by Nie and Yang (2014).Moreover, *trnS* and *trnL* had double copies, while other tRNAs had not. The two rRNA genes of *D. dimidiatus* ab. *rosea* were 771 bp (*rrnS*) and 1,273 bp (*rrnL*) in size. The control regionwas located between the *trnI* and *rrnS* genes with 1297 bp in length, while the overlapping sequences ranged from 1 to 35 bp in 18 regions, and the longest was between *trnK* and *cox2*.The Bayesian inference (BI) tree and IQ phylogenetic tree showed that Anobiidae and Bostrichidae consisted a sister group (Figure 1). The Dermestidae merged with its branches, indicating that Anobiidae was a polyphyletic group, while Bostrichidae and Dermestidae were monophyletic groups. This confirms some previous studies (Karagozlu et al. 2017; Karagozlu et al. 2019). For family Dermestidae, the phylogenetic tree showed that *D. dimidiatus* ab. *rosea* and *D. tessellatocollis* formed in a clade that was a sister group to (*Dermestes maculatus* + *Dermestes frischii*).

**Figure 1.**
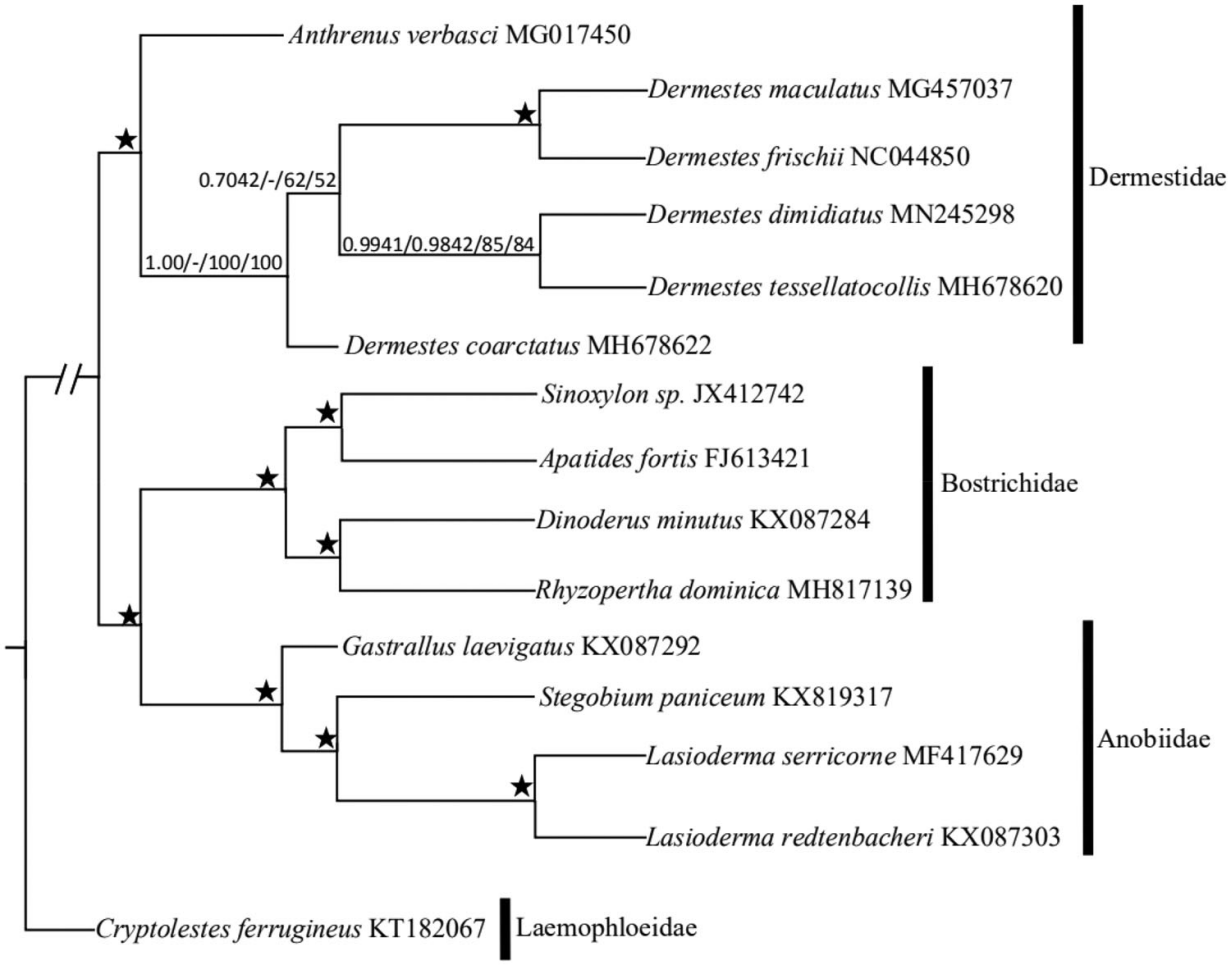
Maximum Likelihood (ML) and Bayesian inference (BI) phylogenetic tree inferred from mitochondrial genomes of superfamily Bostrichoidea species based on two datasets: (a) PCG123; (b) PCG123 and 2 rRNAs. Values above the nodes represented PCG123 Bayesian posterior probabilities/PCG123 and 2 rRNAs Bayesian posterior probabilities/PCG123 bootstrap values/PCG123 and 2 rRNAs bootstrap values. ‘–’ indicates not support, ‘★’ indicated posterior probabilities = 1.00 or ML bootstrap = 100 in all trees.

## Data Availability

Supplemental data for this article is available online at https://www.ncbi.nlm.nih.gov/ with access number MN245298.
